# COVID-19 and Influenza Booster Vaccination Elicits Robust Antibody Responses in Patients with Primary Brain Tumors Comparable to Healthy Adults

**DOI:** 10.3390/cancers18030494

**Published:** 2026-02-02

**Authors:** Teresa Schmidt, Mirko Trilling, Vu Thuy Khanh Le-Trilling, Lucia Asar, Melanie Fiedler, Christoph Oster, Jana Grieger, Giorgio Cappello, Kathrin Kizina, Leandra Grzib, Leon Jekel, Björn Scheffler, Ulrich Sure, Yahya Ahmadipour, Laurèl Rauschenbach, Christoph Kleinschnitz, Ulf Dittmer, Martin Glas, Sied Kebir

**Affiliations:** 1 Division of Clinical Neuro-Oncology, Department of Neurology and Center for Translational Neuro- and Behavioral Sciences (C-TNBS), University Hospital Essen, University of Duisburg-Essen, 45147 Essen, Germany; 2 Institute for Virology, University Hospital Essen, University of Duisburg-Essen, 45147 Essen, Germany; mirko.trilling@uk-essen.de (M.T.);; 3 Institute for the Research on HIV and AIDS-Associated Diseases, University Hospital Essen, University of Duisburg-Essen, 45147 Essen, Germany; 4 DKFZ-Division of Translational Neurooncology at the West German Cancer Center (WTZ), University Medicine Essen, University of Duisburg-Essen, 45147 Essen, Germany; 5 German Cancer Consortium (DKTK), Partner Site Essen-Düsseldorf, Partnership Between DKFZ and University Hospital Essen, 45147 Essen, Germany; 6 German Cancer Research Center (DKFZ), 69120 Heidelberg, Germany; 7 Department of Neurosurgery and Spine Surgery, University Hospital Essen, 45147 Essen, Germany; 8 Department of Neurology and Center for Translational Neuro- and Behavioral Sciences (C-TNBS), University Hospital Essen, University of Duisburg-Essen, 45147 Essen, Germany; 9 Department of Neurology and Neurooncology, St. Marien Hospital, 44534 Lünen, Germany; 10 Department of Neurooncology, Center for Neurology, University Hospital Bonn, University of Bonn, 53127 Bonn, Germany

**Keywords:** COVID-19 booster, influenza booster, primary brain tumor patients, glioma, healthy control, immunogenicity, humoral antibody response

## Abstract

**Background**: Patients with primary brain tumors face profound disease- and treatment-related immunosuppression, placing them at high risk for severe infections. Their capacity to mount protective immune responses to vaccination, particularly to repeated antigen exposures such as COVID-19 boosters, remains critically under-defined, leading to uncertainty in clinical practice. **Methods**: In this prospective cohort study, we analyzed humoral responses to seasonal COVID-19 (mRNA-based) and influenza vaccination in 17 patients with primary brain tumors (recruited from an initial cohort of 37) who received a booster shot, and 19 healthy controls. Serum samples were collected before (T1) and 30 ± 2 days after (T2) vaccination to quantify SARS-CoV-2 anti-Spike (S) IgG and anti-Influenza IgG titers. **Results**: Despite ongoing chemotherapy in 47% of patients, baseline anti-S antibody titers were comparable between groups. Following the booster, median anti-S titers increased significantly and to a similar magnitude in both patients (from 5030 to 18,500 BAU/mL; IQR: 13,885–24,420) and controls (from 4429 to 20,200 BAU/mL; IQR: 11,075–26,680; *p* = 0.6137, Mann–Whitney U test). Only two heavily pre-treated patients showed no booster response. All participants showed sero-positivity to Anti-Influenza IgG at baseline. For the primary brain tumor cohort, a significant increase for anti-Influenza IgG at T2 was observed (*p* = 0.0002). Mean antibody titers did not differ between both cohorts. **Conclusions**: Our findings provide evidence that patients with primary brain tumors can mount robust recall immunity to mRNA vaccines, addressing clinical uncertainty about booster efficacy in this population. These data provide a strong rationale for prioritizing booster vaccinations in this vulnerable population and argue for the inclusion of these patients in future pivotal vaccine trials.

## Simple Summary

Patients with primary brain tumors experience substantial disease- and treatment-related immunosuppression. This study addresses a critical gap in understanding vaccine efficacy in this population. In this prospective cohort study, we evaluated serum antibody responses before and after seasonal mRNA COVID-19 and influenza booster vaccination in patients with primary brain tumors and healthy controls. The results demonstrate robust immune responses to the mRNA COVID-19 and seasonal influenza booster, comparable to those observed in healthy controls, indicating preserved recall immunity. Our findings provide a rationale to prioritize vaccination in this vulnerable population. The data challenge the notion that profound immunosuppression precludes effective recall immunity and support the inclusion of patients with primary brain tumors in future vaccine trials.

## 1. Introduction

Since their introduction, COVID-19 vaccines have been very safe and highly effective in reducing transmission, hospitalizations, and mortality [1,2,3,4,5,6]. However, vaccine-induced immunity especially in terms of sterile immunity wanes after the basic vaccination regime [7,8]. This is particularly relevant upon the occurrence of viral immune escape variants [9,10,11]. Patients with solid tumors and immunocompromised individuals are among those at greatest risk for severe COVID-19 [8,12]. Although initially excluded from pivotal vaccine trials, subsequent studies demonstrated that cancer patients can elicit an immune response, albeit with lower seroconversion rates and reduced antibody titers compared to healthy individuals [12,13,14,15]. However, only one prospective study has assessed the immunogenicity of the BNT162b2 vaccine in patients with primary brain tumors, reporting an 88% seroconversion rate after the second dose, although antibody levels were lower than in healthy controls and were negatively impacted by corticosteroid use [16]. German national guidelines recommend three SARS-CoV-2 antigen exposures (either by vaccination or infection) as baseline immunization for all adults plus additional annual booster doses (to be applied in the autumn using a vaccine adjusted to the circulating SARS-CoV-2 variant) advised for high-risk groups including patients with active neoplastic diseases [17]. The WHO recommends revaccinations 6–12 months after the most recent dose for all individuals who are immunosuppressed [18]. However, safety and efficacy of booster vaccinations in patients with primary brain tumors remained unclear, as they have largely been neglected in recent immunogenicity studies.

This knowledge gap creates a significant clinical dilemma. Given the profound immunosuppression, it is widely assumed that booster immunogenicity might be severely blunted. We hypothesized, however, that established immunological memory in these patients remains sufficiently intact to elicit a robust recall response following an mRNA vaccine booster. To address this, we conducted a prospective study to quantify the humoral immune response to seasonal COVID-19 and influenza vaccination in patients with primary brain tumors and compared it to that of healthy controls.

## 2. Materials and Methods

### 2.1. Study Population

Between May and September 2023, patients at the Division of Neuro-Oncology, Essen University Hospital, were offered to participate in the study. A structured survey captured data on COVID-19 vaccination history, prior SARS-CoV-2 infections, and willingness to receive a booster in accordance with German guidelines.

Eligible participants met the following criteria: (1) adults with a primary brain tumor, (2) complete COVID-19 immunization (≥3 antigen exposures, including ≥2 vaccinations), and (3) ≥12 months since the last antigen exposure (COVID-19 vaccine or SARS-CoV-2 vaccine dose). The control group consisted of healthy healthcare workers and relatives without chronic illnesses or immunosuppressive therapy. The study was approved by the local ethics committee (22-10519-BO).

### 2.2. Vaccination and Blood Sampling

Participants provided informed consent and underwent blood sampling at two timepoints: T1 (Baseline): Before vaccination, blood was collected for complete blood count, T-/B-cell quantification (patients only), anti-SARS-CoV-2 spike (Anti-S), and Influenza A/B antibody titers; T2 (Follow-up): 30 ± 2 days post-vaccination, a second blood sample was taken for Anti-S and Influenza A/B antibody titers. All participants received a seasonal COVID-19 booster (Comirnaty Omikron XBB.1.5; BioNTech Manufacturing GmbH, Mainz, Germany) and influenza vaccine (Influvac Tetra 23/24; Bad Homburg, Germany) after T1.

### 2.3. Antibody Testing

Serum samples were stored at 4 °C for a maximum of 5 days, centrifuged at 3500 *g* for 15 min, and stored at −20 °C until further analyses. Quantification of anti-SARS-CoV-2 binding antibody titers was conducted using the diagnostic LIAISON SARS-CoV-2 TrimericS IgG assay (Diasorin; Saluggia, Italy) according to manufacturer’s instructions. Relative light units (RLUs) were automatically converted into quantitative values for binding antibody units (BAU/mL). Samples reaching more than 2080 BAU/mL (detection limit of the assay) were diluted with phosphate-buffered saline and subsequently retested. Values equal to or greater than 33.8 BAU/mL are considered positive. For the quantification of anti-Influenza virus IgG, an indirect in-house ELISA was performed. Briefly, MaxiSorp microtiter plates (Nunc; Waltham, MA, USA) were coated with Influvac Tetra 2024/2025 (Viatris; Canonsburg, PA, USA) in ELISA binding buffer (0.1 M Na2HPO4, pH 9.0). Plates were incubated overnight at 4 °C. Non-specific binding sites were blocked with a solution of 10% (*v*/*v*) FCS in PBS for 1 h at room temperature. Wells were washed two times with ELISA wash buffer (PBS with 0.1% [*v*/*v*] Tween-20) before serum samples were added and incubated overnight at 4 °C. After three washing steps with ELISA wash buffer, peroxidase-labeled secondary antibody (A2290, Sigma; Darmstadt, Germany) was added and incubated for 1 h at room temperature. After four washing steps, the calorimetric peroxidase reaction was started using TMB substrate. The reaction was stopped with 0.5 M HCl before the absorbance was quantified using a microplate multireader (Mithras LB 943; Berthold; Bad Wildbad, Germany). For the conversion of optical density (OD) values into arbitrary Influenza virus-specific IgG units, reference samples were applied to all plates and compared to the 1:1250 and 1:6250 dilutions of the serum samples to be analyzed. The ratio of the OD of the corresponding reference and the serum samples were calculated in percent. The arbitrary units reflect the mean value of the percent values of the 1:1250 and 1:6250 dilutions of the serum samples.

### 2.4. Statistical Analysis

Data were analyzed using GraphPad Prism (v9.0) and R (v4.1). Due to the sample size and potential for non-normal data distribution, non-parametric tests were used for group comparisons. The Mann–Whitney U test was used for unpaired comparisons (e.g., patient vs. control titers at T2), and the Wilcoxon matched-pairs signed-rank test was used for paired comparisons (e.g., T1 vs. T2 within a group). A *p*-value of <0.05 was considered statistically significant. This was an exploratory study; no formal power calculation was performed. Exploratory analyses were performed to investigate potential associations between clinical parameters and a high-titer response (defined as >15,000 BAU/mL), acknowledging the limited statistical power of these analyses.

#### Exploratory Analyses and ROC Curves

To explore potential predictors of a strong humoral response, we defined a high-titer response as anti–S IgG at T2 > 15,000 BAU/mL. Candidate predictors were selected a priori based on clinical availability and biological plausibility (sex, age at booster, and time since last SARS-CoV-2 antigen exposure, and, in glioma patients, absolute lymphocyte count at T1). We fitted multivariable logistic regression models (binomial family) and generated ROC curves using model-predicted probabilities across all thresholds; AUC was computed in R (pROC). Model 1 included glioma patients only (sex, age, time since last antigen exposure, lymphocyte count at T1). Model 2 included all participants (sex, age, cohort [glioma vs. control], time since last antigen exposure). These analyses were exploratory, and hypothesis-generating given the sample size.

## 3. Results

### 3.1. Study Population

**Figure 1:**
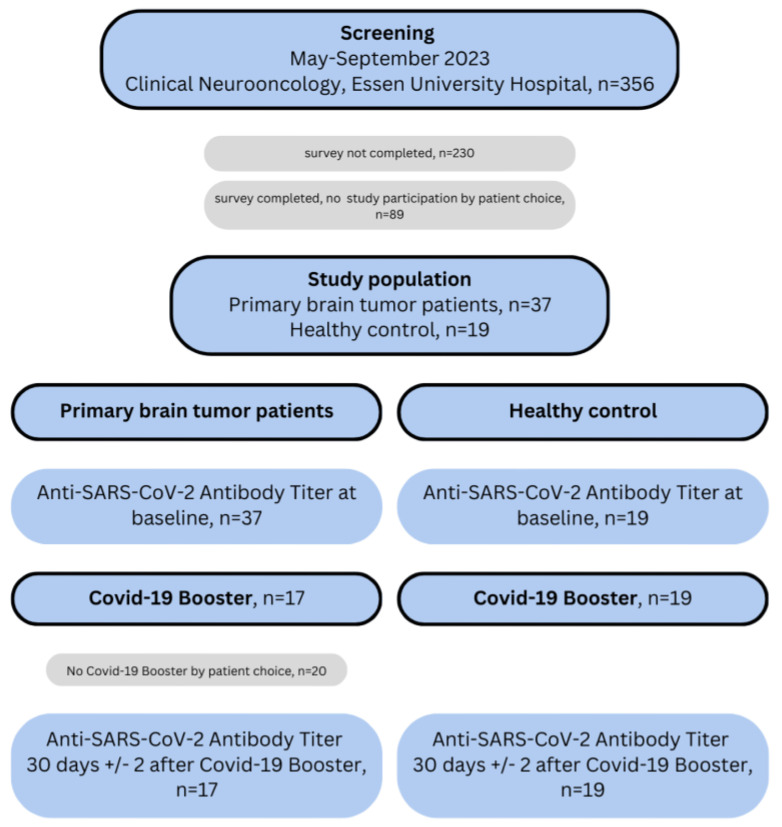
Schematic overview of the study design.

Of 356 patients contacted, 126 (35%) completed the COVID-19 vaccination survey. Among respondents, 122 (97%) reported complete immunization with at least three SARS-CoV-2 antigen exposures, and 4 (3%) reported to be unvaccinated. A total of 37 patients with primary brain tumors underwent baseline Anti-S antibody testing (T1, Figure 1). At baseline, 22 patients (60%) were receiving concurrent chemotherapy, and 5 (14%) were on oral corticosteroids. The median time since the last COVID-19 vaccination was 22 months (interquartile range [IQR], 18–23; Table 1). Seventeen patients (46%) received the COVID-19 booster and the seasonal influenza vaccine (glioma group, Figure 1). Reasons for not receiving a booster included patient preference (*n* = 16), recent infection or vaccination (*n* = 2), and clinical deterioration (*n* = 2). Among the glioma group, 8 patients (47%) were undergoing concurrent chemotherapy (Table 2). SARS-CoV-2 sero-positivity at baseline was observed in 36 of 37 patients (97%). The control group comprised 19 healthy participants, with a median age of 55 years and a balanced sex distribution. The median time since their last COVID-19 vaccination was 18 months (IQR, 13–20; Table 1). As expected, all controls demonstrated SARS-CoV-2 sero-positivity at baseline.

### 3.2. COVID-19 Booster Response

**Figure 2:**
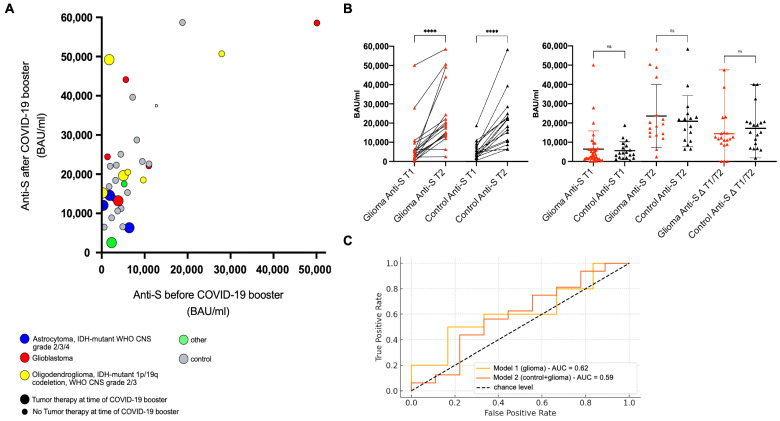
Immune Response to COVID-19 Vaccination in Glioma Patients and Controls. (**A**) Scatter plot of anti-spike (anti-S) antibody titers before and after COVID-19 booster in the glioma group and control. (**B**) Paired (**left**) and unpaired (**right**) comparison of anti-S titers pre- and post-booster, including overall change in antibody levels. (**C**) Receiver operating characteristic (ROC) curve predicting anti-S titers at T2 in glioma patients based on gender, age at booster, time since last antigen exposure, and absolute lymphocyte count at T1 (Model 1, yellow and ROC curve predicting a high anti-S titer (defined as >15,000 BAU/mL) across the study population, incorporating gender, age, and group status (glioma vs. control; Model 2, orange). Ns = non-significant. **** = *p* < 0.0001.

In the glioma group, median Anti-S antibody levels increased significantly from 5030 BAU/mL (IQR, 1850–6408) at T1 to 18,500 BAU/mL (IQR, 13,885–24,420) at T2. Two patients showed no measurable increase in antibody response (Figure 2A,B); both underwent treatment at the time of blood sampling, including chemotherapy, and showed no evidence of high-grade myelotoxicity.

In the control group, median Anti-S levels rose from 4429 BAU/mL (IQR, 2121–7675) at T1 to 20,200 BAU/mL (IQR, 11,075–23,680) at T2, with a comparable pattern of response. There were no significant differences between groups at T1 (*p* = 0.7175), T2 (*p* = 0.6137), or in the overall change (*p* = 0.4494) (Figure 2A,B), indicating that the booster immune response to an mRNA-based COVID-19 vaccine is similar in glioma patients compared to healthy controls.

In an exploratory analysis, we investigated potential predictors of a high-titer response (>15,000 BAU/mL). We did not identify any clear associations for clinical or laboratory parameters, including gender, age at booster administration, time since last antigen exposure, or absolute lymphocyte count at T1. However, these analyses were not powered to definitively assess such predictors (Figure 2C).

### 3.3. Seasonal Influenza Vaccination Response

**Figure 3:**
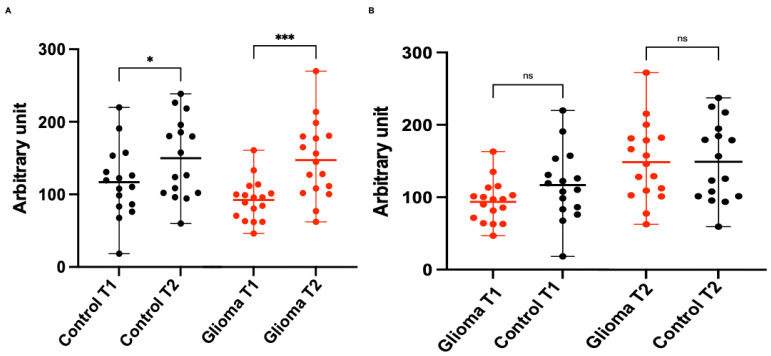
Immune Response to seasonal Influenza Vaccination in Glioma Patients and Controls. (**A**) Paired influenza antibody levels pre- and post-booster for both glioma and control groups. (**B**) Unpaired comparison influenza antibody levels at T1, T2. Ns = non-significant. * = *p* < 0.05. *** = *p* < 0.001.

Seasonal influenza vaccines were co-administered with COVID-19 boosters in both groups. At T1, all glioma patients showed measurable sero-positivity for Anti-Influenza Virus IgG. At T2, a significant detectable change in IgG Anti-Influenza Virus was seen in 16 (94%) glioma patients (*p* = 0.0002; Figure 3). In the control group, T1 sero-positivity rates were detected in all patients for Anti-Influenza Virus IgG (Figure 3) and a positive change was measured in 10 control patients (63%) at T2 (*p* = 0.0739; Figure 3). There were no significant differences for Anti-Influenza Virus IgG Titer between glioma and control groups at T1 and T2.

### 3.4. Primary Brain Tumor Patient Cohort

A baseline Anti-S assessment for the primary brain tumor cohort was performed for 37 patients. Within this cohort, 22 (60%) patients received concurrent chemotherapy, and 5 (14%) patients received concurrent dexamethasone treatment. Comparing Baseline Anti-S titers between patients with (17 (46%)) and without (20 (54%)) subsequent Covid-Booster showed median Anti-S levels of 5030 BAU/mL (IQR 1850–6408) and 2480 BAU/mL (IQR 1360–6555; *p* = 0.5052), respectively. A subgroup analysis within the glioma group comparing individuals with (*n* = 8) and without (*n* = 9) concurrent chemotherapy did not reveal statistically significant differences in antibody responses to the COVID-19 booster and influenza vaccine (Table 2). There was no significant difference in mean Baseline Anti-S titers between primary brain tumor patients with and without dexamethasone (*p* = 0.0654).

Given the very small sample sizes, these analyses were not powered to detect modest effects, and no firm conclusions can be drawn regarding the impact of concurrent chemotherapy.

## 4. Discussion

In this prospective study, we demonstrate that patients with primary brain tumors, despite their profound disease- and treatment-related immunosuppression, can mount a robust humoral recall response to an mRNA-based COVID-19 booster and inactivated influenza vaccine. The magnitude of this response was remarkably comparable to that of healthy controls, providing important reassurance for vaccination strategies in this vulnerable population. Interestingly, the glioma group showed a stronger immune response to the influenza vaccine compared to the control group.

Our data address a critical clinical question. To our knowledge, this is the first study to evaluate the serological response to a seasonal COVID-19 and influenza booster in previously vaccinated patients with primary brain tumors. With regard to the mRNA vaccine, our data suggest that immunological recall capacity may remain well-preserved even after a median of 22 months following the last documented antigen exposure. While previous studies in patients with solid tumors reported generally preserved immunity [12,13], our data extend these observations to a uniquely immunosuppressed population. Critically, whereas a prior study on primary vaccination in brain tumor patients elicited lower antibody levels compared to controls [16], our data suggest that a booster dose can overcome this initial deficit, generating antibody titers comparable to those in healthy individuals. This implies that while the establishment of de novo immunity may be impaired, immunological memory, once established, remains highly functional and can be effectively recalled. The absence of identifiable clinical or laboratory predictors for high post-booster titers echoes previous observations in diverse oncologic cohorts [16], highlighting an intrinsic, yet-to-be-defined patient-specific variability.

When comparing the glioma cohort with the control group following administration of the inactivated influenza vaccine—which predominantly elicits a humoral immune response—the mean baseline anti-influenza IgG titers were lower in patients with glioma. Notably, however, 30 days post-vaccination, the glioma group demonstrated a more pronounced immune response, resulting in comparable mean anti-influenza IgG titers at T2 between the two groups. At least within this small study cohort, we did not observe a significant difference in the vaccine-induced immune response in patients with glioma, in contrast to previously published data [19]. This observation suggests that, even in a heavily pretreated glioma population, a robust vaccine-induced humoral immune response can be achieved despite prior exposure or recall immunity.

One of the most notable findings of this study is the strong humoral immune response elicited by the mRNA-based SARS-CoV-2 booster and the inactivated seasonal influenza vaccine. These results underscore the capacity of distinct vaccine platforms to induce robust immune responses in immunocompromised individuals. The potential superiority of mRNA vaccines in overcoming immune hyporesponsiveness remains uncertain and requires further investigation.

Our study has several important limitations that must be acknowledged upfront. First and foremost, the findings are constrained by a significant potential for selection bias. Of 356 patients initially screened, only 17 (4.8%) were ultimately included in the booster analysis. This low participation rate suggests our cohort likely represents a healthier, more motivated subgroup, which may limit the generalizability of our optimistic findings to the broader, more frail population of brain tumor patients. Second, the small sample size (*n* = 17) means the study was not powered for subgroup analyses and the results should be considered hypothesis-generating, requiring validation in larger, multi-center cohorts. Third, our immunological assessment was limited to serological binding antibody titers. We did not measure functional antibody responses, such as virus neutralization capacity, or T-cell-mediated cellular immunity, which are critical for a complete understanding of protective immunity. Finally, our cohort primarily included patients with gliomas, and findings may not extrapolate to other brain tumor subtypes.

In conclusion, our findings carry significant clinical implications. They provide a strong, albeit preliminary, rationale for the proactive administration of COVID-19 mRNA and seasonal influenza vaccine boosters in patients with primary brain tumors, even in the context of ongoing chemotherapy. Our work challenges the rationale for the broad exclusion of this population from vaccine trials. For non-replicating platforms such as mRNA vaccines, their inclusion is not only ethically appropriate but scientifically essential to generate the evidence needed to protect this vulnerable group.

## 5. Conclusions

Patients with primary brain tumors, including those on active therapy, demonstrate a robust humoral recall response to mRNA-based COVID-19 boosters, achieving antibody levels comparable to healthy controls. These findings support prioritizing booster vaccinations in this vulnerable population.

## Figures and Tables

**Table 1:** Study group characteristics.

Characteristic	Primary Brain Tumor (*n* = 17)	Healthy Control (*n* = 19)	*p* Value
***Age (y), median (IQR)***	55 (46–63)	55 (32–61)	0.4370 ^2^
***Gender, n (%)***			0.5251 ^1^
*Male*	10 (59)	9 (47)	
*Female*	7 (41)	10 (53)	
***Time since last COVID-19 vaccination (m), median (IQR)***	22 (18–23)	18 (13–20)	0.0554 ^2^
***Anti-SARS-CoV-2 Antibody Titer at baseline (BAU/mL), median (IQR)***	5030 (1850–6408)	4429 (2121–7675)	>0.9999 ^2^
***Anti-SARS-CoV-2 Antibody Titer at 30 days +/− 2 after COVID-19 Booster***	18,500 (13,885–24,420)	20,200 (11,075–23,680)	0.9858 ^2^
***Anti Influenza Titer at baseline (Arbitrary units), median (IQR)***	97 (72–103)	115 (86–137)	0.0872 ^2^
***Anti Influenza Titer at 30 days +/− 2 after seasonal Influenza Booster, (Arbitrary units), median (IQR)***	147 (110–183)	142 (102–188)	0.9858 ^2^

Abbreviations: IQR: interquartile range; m: months; *n*: number; y: year; ^1^ Fisher exact test; ^2^ Mann–Whitney Test.

**Table 2:** Primary brain tumor group characteristics.

Characteristic	Primary Brain Tumor with COVID-19 Booster (*n* = 17)	Primary Brain Tumor Without COVID-19 Booster (*n* = 20)	*p* Value
***Diagnosis, n (%)***			
*Glioblastoma*	5 (29)	9 (45)	
*Astrocytoma, IDH-mutant WHO CNS grade 2–4*	4 (24)	6 (30)	
*Oligodendroglioma, IDH-mutant 1p/19q codeletion, WHO CNS grade 2–3*	6 (35)	3 (15)	
*other*	2 (12)	2 (10)	
***MGMT promoter methylation status***			0.7120 ^1^
*Methylated*	9 (53)	12 (60)	
*Unmethylated*	6 (35)	5 (25)	
*Unknown*	2 (12)	3 (15)	
***Treatment line, n (%)***			
*Primary*	8 (47)	10 (50)	
*Secondary*	6 (35)	4 (20)	
*Tertiary or more*	3 (18)	6 (30)	
***Concurrent treatment***			0.0819 ^1^
*Chemotherapy*	8 (47)	15 (75)	
*TTFields therapy*	0	1 (5)	
*No treatment*	9 (53)	4 (20)	
***Concurrent dexamethasone therapy***			0.0498 ^1^
*yes*	0 (0)	5 (25)	
*no*	17 (100)	15 (75)	
***Time (m) from Tumor diagnosis to T1, median (IQR)***	29 (10–49)	24 (10–67)	0.0897 ^2^
***Anti-SARS-CoV-2 Antibody Titer (BAU/mL), median (IQR)***			
*Before COVID-19 booster*	5030 (1850–6408)	2480 (1360–6555)	0.5052 ^2^

Abbreviations: CNS: central nervous system; IDH: isocitrate dehydrogenase; IQR: interquartile range; m: months; MGMT, O-6-methylguanine-DNA methyltransferase; *n*: number; T1: time of blood sampling at baseline; WHO: World Health Organisation; ^1^ Fisher exact test; ^2^ Mann–Whitney Test.

## Data Availability

All data supporting the findings of this study are available within the article file and from the corresponding author upon reasonable request.
